# Larger lesion volume in people with multiple sclerosis is associated with increased transition energies between brain states and decreased entropy of brain activity

**DOI:** 10.1162/netn_a_00292

**Published:** 2023-06-30

**Authors:** Ceren Tozlu, Sophie Card, Keith Jamison, Susan A. Gauthier, Amy Kuceyeski

**Affiliations:** Department of Radiology, Weill Cornell Medicine, New York, NY, USA; Horace Greeley High School, Chappaqua, NY, USA; Judith Jaffe Multiple Sclerosis Center, Weill Cornell Medicine, New York, NY, USA; Department of Neurology, Weill Cornell Medical College, New York, NY, USA; Brain and Mind Research Institute, Weill Cornell Medicine, New York, NY, USA

**Keywords:** Multiple sclerosis, Brain activity dynamics, Network control theory, Entropy

## Abstract

Quantifying the relationship between the brain’s functional activity patterns and its structural backbone is crucial when relating the severity of brain pathology to disability in multiple sclerosis (MS). Network control theory (NCT) characterizes the brain’s energetic landscape using the structural connectome and patterns of brain activity over time. We applied NCT to investigate brain-state dynamics and energy landscapes in controls and people with MS (pwMS). We also computed entropy of brain activity and investigated its association with the dynamic landscape’s transition energy and lesion volume. Brain states were identified by clustering regional brain activity vectors, and NCT was applied to compute the energy required to transition between these brain states. We found that entropy was negatively correlated with lesion volume and transition energy, and that larger transition energies were associated with pwMS with disability. This work supports the notion that shifts in the pattern of brain activity in pwMS without disability results in decreased transition energies compared to controls, but, as this shift evolves over the disease, transition energies increase beyond controls and disability occurs. Our results provide the first evidence in pwMS that larger lesion volumes result in greater transition energy between brain states and decreased entropy of brain activity.

## Author Summary

We investigated the brain-state dynamic and energy landscapes in healthy individuals and people with multiple sclerosis (pwMS). We also investigated the entropy of brain activity and its association with transition energy between brain states and lesion volume. We clustered regional brain activity time series to identify the brain states. Then, we applied network control theory using structural connectivity network to identify the minimum required energy to transition between brain states. We observed the pwMS without disability showed decreased transition energy, while pwMS with evidence of disability showed increased transition energy compared to healthy individuals. Lower entropy of brain activity was associated with greater lesion load and larger transition energy. This study provides a possible mechanism of how MS-related damage to the brain’s structural backbone can impact brain dynamics, entropy, and energetics.

## INTRODUCTION

Multiple sclerosis (MS) is a chronic disease characterized by neuroinflammation and, eventually, neurodegeneration in the central nervous system (CNS). The size and location of the lesions in the CNS are very heterogeneous (Barkhof, 2002), resulting in different patterns of structural damage among people with MS (pwMS). Structural damage to the brain may result in changes to its functional activity patterns (Tommasin et al., 2018); however, how different patterns of structural damage can modify dynamics of functional activity have not been fully characterized in pwMS.

Functional MRI (fMRI) measures functional brain activity patterns over time, information which can be used to quantify brain activity dynamics or to estimate patterns of regional co-activation, that is, functional connectivity (FC). Both increased or decreased fMRI-based activity/connectivity has been observed in pwMS, where increases are often interpreted as a compensatory mechanism serving to limit clinical disability (Buyukturkoglu et al., 2020; Filippi et al., 2013; Sbardella et al., 2015). FMRI in pwMS has largely been used to investigate the association between the brain’s static and/or dynamic FC and motor/cognitive impairment. (Buyukturkoglu et al., 2020; Tozlu et al., 2021a; Tozlu et al., 2021b). Most FC studies in pwMS have been static in nature (Filippi et al., 2013; Sbardella et al., 2015), ignoring shorter scale changes in FC that have been shown to occur (Calhoun et al., 2014). These shorter scale changes in FC can be captured via dynamic FC (dFC) analysis that involves clustering FC matrices computed via sliding windows of the fMRI time series data (Allen et al., 2014). This approach was previously used to differentiate between the pwMS and healthy controls (HC) (Leonardi et al., 2013; Rocca et al., 2019; Tozlu et al., 2021a), to compare the dynamics between cognitively impaired versus preserved pwMS (d’Ambrosio et al., 2019), and to investigate the relationship between alterations in dFC dynamics and cognition in MS (Fuchs et al., 2022; van Geest et al., 2018a; van Geest et al., 2018b). However, calculating dFCs with a sliding-window approach is not straightforward since the estimation of the window length and shift size of the sliding windows is required; moreover, correlations are still used to describe coactivations of pairs of regions as in static FC. As an alternative approach, clustering can be directly applied to the time series of regional activity to define distinct brain activity states. This approach was successfully used in HC to identify brain activity states that occur while performing a working memory task, quantify the effects of psychedelics, and identify changing dynamics of activity patterns in stroke patients (Cornblath et al., 2020; Olafson et al., 2022; Singleton et al., 2022). In addition to the identification of the brain’s dynamic states, the same studies used network control theory (NCT) approach (Gu et al., 2015; Stiso et al., 2019; E. Tang et al., 2017) to identify the minimum energy required to transition between these dynamic brain states. However, no study to date has applied brain activity clustering or NCT in a population of pwMS, let alone investigate differences in brain dynamics and energetics across disability subgroups in MS.

In terms of capturing brain activity dynamics, entropy is also an important metric with which to quantify the amount of regularity/unpredictability in brain activity time series (Pincus, 1991). Higher entropy of brain activity has been associated with a larger repertoire of available brain states (Carhart-Harris et al., 2014), and decreased NCT-based transition energy under psychedelics compared to placebo was found to be associated with greater increases in entropy of brain states (Singleton et al., 2022). Studies in disease/disorders have shown (a) greater entropy in controls compared to people with attention-deficit/hyperactivity disorder (ADHD) as well as a correlation between lower entropy and more severe ADHD symptom scores (Sokunbi et al., 2013) and (b) decreased entropy in regions containing a stroke lesion and their contralesional hemisphere homologues (Saenger et al., 2018). One study in relapsing-remitting MS (RRMS) showed entropy was associated with an individual’s disability level, where more disability was related to increased entropy (Sokunbi et al., 2013; Zhou et al., 2016). However, no study to date has investigated the link between the entropy of brain activity and (a) lesion volume or (b) NCT-based transition energy between brain states in MS.

In this study, we first aimed to identify recurrent dynamic brain states in both HC and pwMS by unsupervised clustering of regional resting-state fMRI activity. Note that, although related (see Olafson et al., 2022), this approach differs from dFC (sliding-window FC) in that instead of clustering dFC matrices we are clustering the brain activity vectors from each TR to identify commonly occurring patterns of brain activation (not FC). We calculated metrics quantifying the dynamics of these brain activity states, including transition probability and fractional occupancy, and used NCT and individuals’ structural connectomes (SC) to compute the energy required to transition between pairs of brain states. We hypothesized that greater energy is required for brain-state transitions in pwMS with disability compared to both controls and pwMS with no evidence of disability. Finally, we investigated the association between overall transition energy and the information contained in the brain activity signal, as captured by entropy. We calculated the entropy of each region’s activity over the entire fMRI scan and summarized the global entropy by taking the average of the regional measures. Our hypothesis was that decreased global entropy is associated with both increased transition energy and lesion load. This paper is the first to quantify MS-related shifts in the brain’s energetic landscape and to link entropy/energetic demand of brain activity to lesion burden in pwMS.

## MATERIAL AND METHODS

### Subjects

One hundred pwMS (age: 45.5 [36.7, 56.0], 66% females) with a diagnosis of clinically isolated syndrome (CIS)/MS (CIS = 7, RRMS = 88, primary/secondary progressive primary progressive MS [PPMS] and secondary progressive MS [SPMS] = 5) and 19 HC (age: 45 [35, 49], 55% females) were included in our study. All MS subgroups were included in the analyses as we wanted to investigate these imaging biomarkers across the spectrum of disability severities, which are often related to disease subtype. All subjects who had preprocessed imaging data available included in our study. MRIs and demographic data were collected (age, sex, and race for both HC and pwMS, clinical phenotype and disease duration for pwMS). The expanded disability status scale (EDSS) was used to quantify disability in pwMS, where an EDSS of 2 was used as a threshold to categorize the pwMS into no disability (EDSS < 2) or evidence of disability (EDSS ≥ 2) groups. An EDSS of greater than or equal to 2 is the cutoff for clinical definitions of “disability,” whereas scores below that are not considered to be disabled and scores above it correspond to minimal, moderate, or severe disability. All studies were approved by an ethical standards committee on human experimentation and written informed consent was obtained from all patients. Participants were excluded if they had contraindications to MRI, and controls were further excluded if they had ever been diagnosed with or were currently on medication for a neurological or psychological disorder.

### Image Acquisition, Processing, and Structural Connectome Extraction

MRI data were acquired on a 3T Siemens Skyra scanner (Siemens, Erlangen, Germany) with a 20-channel head-neck coil. Anatomical MRI (T1/T2/T2-FLAIR, 1-mm^3^ isovoxel), resting-state fMRI (6 min, TR = 2.3 s, 3.75 × 3.75 × 4 mm voxels) and diffusion MRI (55 directions HARDI, b = 800, 1.8 × 1.8 × 2.5 mm voxels) acquisitions were performed. Multi-echo 2D GRE field maps were collected for use with both fMRI and diffusion MRI (0.75 × 0.75 × 2 mm voxels, TE1 = 6.69 ms, ΔTE = 4.06 ms, number of TEs = 6).

White matter (WM) and gray matter (GM) were segmented and GM further parcellated into 86 FreeSurfer-based regions (68 cortical and 18 subcortex/cerebellum) (Fischl & Dale, 2000) and a fine-grained atlas cc400 (321 cortical and 71 subcortex/cerebellum) using FreeSurfer (Craddock et al., 2012). The white and gray matter surfaces were manually checked and hand edited with control points and reconstruction editing if necessary. As described elsewhere (Kuceyeski et al., 2016), fMRI preprocessing included simultaneous nuisance regression and removal of WM and cerebrospinal fluid (CSF) effects (Hallquist et al., 2013), followed by high-pass filtering (≥0.008) using the CONN v18b toolbox (Whitfield-Gabrieli & Nieto-Castanon, 2012) and SPM12 in Matlab. Nuisance regressors included 24 motion parameters (6 rotation and translation, temporal derivatives, and squared version of each) and the top 5 eigenvectors from eroded masks of both WM and cerebro-spinal fluid. The mean fMRI signal over all voxels in each of the 86 regions for each TR was calculated to obtain a regional time series matrix of BOLD activity.

Diffusion MRI was interpolated to isotropic 1.8-mm voxels, and then corrected for eddy current, motion, and EPI distortion with the eddy command from FSL 5.0.11 (Andersson & Sotiropoulos, 2016) using the outlier detection and replacement option (Andersson et al., 2016). MRtrix3Tissue (https://3tissue.github.io), a fork of Mrtrix3 (Tournier et al., 2019), was used to estimate a voxel-wise single-shell, three-tissue constrained spherical deconvolution model (SS3T-CSD) and then compute whole-brain tractography for each subject. We performed deterministic (sd-stream) tractography with MRtrix3 (Tournier et al., 2010; Tournier et al., 2012) with uniform seeding at each white matter voxel, after which the SIFT2 global filtering algorithm (Smith et al., 2015) was applied to account for bias that exists in greedy, locally optimal streamline reconstruction algorithms. The SC matrix was constructed by taking the sum of the SIFT2 weights of streamlines connecting pairs of regions and then dividing by the sum of the two regions’ volumes.

Lesion masks were produced for each pwMS in a semiautomated process. The WM hyperintensity lesion masks were created by running the T2 FLAIR images through the lesion segmentation tool (LST) and were further hand edited as needed. T2 FLAIR-based lesion masks were transformed to the individual’s T1 native space using the inverse of the T1 to GRE transform and nearest neighbor interpolation. Individual T1 images were then normalized to MNI space using FSL’s linear (FLIRT) and nonlinear (FNIRT) transformation tools (https://www.fmrib.ox.ac.uk/fsl/index.html); transformations with nearest neighbor interpolation were then applied to transform the native anatomical space T2FLAIR lesion masks to MNI space. The transformations were concatenated (T2FLAIR to T1 to MNI) to minimize interpolation effects. Lesions were manually inspected after the transformation to MNI space to verify the accuracy of coregistration and lesion volume (in mm^3^) calculated.

### Brain-State Identification

**Figure 1.**
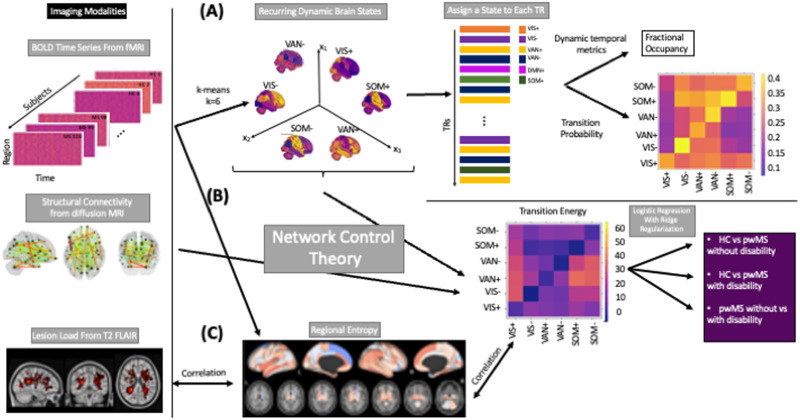
The workflow of the study. (A) Regional BOLD time series for all subjects (19 HC and 99 pwMS) were collected and k-means was applied to identify commonly occurring brain states. This approach allows us to assign a state to each TR in each individual’s scan. Metrics such as fractional occupancy and transition probability for each state were then calculated. (B) Network control theory was applied to compute the minimum energy required to transition between pairs of brain states or to remain in the same state. Logistic regression with ridge regularization was used to predict class (HC, pwMS with no disability, pwMS with disability) using state transition/persistence energy. (C) Entropy of regional BOLD time series were also computed, compared across the three groups and global entropy was associated with global transition energy and global disease burden (log lesion volume).

K-means clustering was used to identify commonly recurring brain states (see Figure 1 for the workflow of the study) over all subjects’ normalized regional brain activity time series. We used both the elbow criterion and the gain in explained variance to identify the optimal number of clusters, which we identified to be six (see Supporting Information Figure S2). Once the optimal number of clusters was identified, clustering was run 10 times. Within each of the 10 runs, clustering was restarted 50 times with different random initializations to identify the final clusters having the best separation, that is, those that minimized the Pearson correlation between pairs of cluster centroids. Adjusted mutual information (AMI) (Nguyen, 2021) was computed to assess the stability of the clustering results over the 10 repetitions; AMI was greater than 0.89 for all pairs of repetitions indicating robust cluster assignment (see Supporting Information Figure S1).

Finally, we replicated the cluster analysis using a higher dimensional atlas (cc400), where optimal k was identified as 5 (see Supporting Information Figure S7). The results obtained with the cc400 atlas were interpreted in the main text and the figures associated with those results were given in the supplementary materials.

Each of the 86 regions was assigned to one of nine networks, including the seven Yeo functional networks, a subcortical and a cerebellar network (Yeo et al., 2011). The network-level contributions of the six centroids were characterized by calculating the cosine similarity of each state’s centroid (positive and negative parts were analyzed separately) and nine binarized vectors representing each of the networks. Because the mean signal from each scan’s regional time series was removed during high-pass filtering, positive values reflect signal intensity above the mean (high-amplitude) and negative values reflect signal intensity below the mean (low-amplitude). The brain states were named by identifying the network with the largest magnitude activation (either high+ or low- amplitude). The following dynamic brain-state metrics were calculated: (a) transition probability between pairs of states and the persistence probability of remaining in the same state and (b) fractional occupancy, defined as the number of TRs assigned to each cluster out of the total number of TRs (see Table 1).

**Table 1.** The MRI-based metrics used in the study

**Metric**	**Definition**	**Interpretation**
**Transition probability**	The probability that a brain switches to a different state or remains in the same state (i.e., persistence probability) at the next time point	A higher transition probability between two states means this transition is more likely to occur than others.
**Fractional occupancy**	Number of TRs assigned to each state out of the total number of TRs	Higher fractional occupancy in a specific state means more time is spent in this state compared to another states.
**Transition energy**	The minimum energy required to transition between pairs of brain states or to remain in the same state over some time	Higher transition energy means the transition requires more energy to complete. Transition energy is highly negatively correlated with transition probability.
**Entropy**	A measure indicating the complexity and predictability of a dynamic system	Higher entropy means increased complexity and decreased predictability of a system.

### Transition Energy Calculations

As previously described (Cornblath et al., 2020; Singleton et al., 2022), NCT can be used to understand how the structural (white matter) connectome constrains dynamic brain-state changes. Specifically, it can be used to compute the minimum energy (*E*_*m*_) required to transition between pairs of brain states or to remain in the same state (see Table 1). To begin, we employed a time-invariant model:

$$\dot{s}\left(t\right)=As\left(t\right)+Bu\left(t\right)$$

where *A* is the structural connectivity matrix with a dimension of 86 × 86, *s*(*t*) is a vector of length 86 containing the regional activity at time *t*, and *u*(*t*) is the external input to the system. In our case, *B* is an identity matrix with a dimension of 86 × 86 that indicates each region has uniform control over the system.

When computing the transition energy (*E*_*m*_) from the initial activity *s*_0_ to final activity *s*_*f*_ over some time *T*, we use an invertible controllability Gramian *W* to control the structural connectivity network *A* from the set of network nodes *B*. The invertible controllability Gramian *W* is defined as

$$W=\int_{0}^{T}e^{A\left(T-\tau \right)}\;B\;B^{tr}\;e^{A^{tr}\left(T-\tau \right)}\;d\tau$$

Optimal *T* was identified by grid searching between 0.001 and 10 and identifying the value that maximized the magnitude of the Spearman correlation between the entries in the transition probability matrix and the entries in the transition energy matrix. The correlation between transition probability and energy is expected to be negative since the brain prefers trajectories through state space requiring minimal input energy given structural constraints, therefore the brain tends to change less between states if the minimum required energy is greater. Here, we identified an optimal *T* of 1, which yielded a correlation of −0.83 (*p* value < 0.001) (see Supporting Information Figure S5), while the optimal *T* was 0.5 for the cc400 atlas with a correlation coefficient of −0.91 (see Supporting Information Figure S12).

After computing the invertible controllability Gramian *W* and optimizing *T*, the transition energy (*E*_*m*_) is then calculated as the quadratic product between the inverted controllability Gramian *W* and the difference between *e*^*AT*^ *s*_0_, which represents the activation at time *T* assuming brain activity spreads in a network diffusion manner over the SC from the initial state *s*_0_, and the final state *s*_*T*_:

$$E_{m}={\left(e^{AT}\;s_{0}-s_{T}\right)}^{tr}\;W^{-1}\;\left(e^{AT}\;s_{0}-s_{T}\right).$$

Since there were six brain states, the transition energy matrix is of size 6 × 6 where off-diagonal entries contain the energy required to transition between each pair of unique states *s*_0_ and *s*_*T*_, where *s*_0_ ≠ *s*_*T*_, and diagonal entries contain persistence energy required to stay in the same state (*s*_0_ = *s*_*T*_).

### Entropy of Brain Activity

To investigate how disease burden (lesion load) and disability level in pwMS were related to the amount of information contained in the brain activity signal, regional entropy was calculated directly on the BOLD time series. Denoting the regional BOLD time series in a region by *s*(*t*) = (*s*(1), …, *s*(*n*)) where *n* is the number of the BOLD time series acquisition. We define two template vectors of length *m* such as *s*_*m*,*i*_ = (*s*(*i*), *s*(*i* + 1), …, *s*(*i* + *m* − 1)) and *s*_*m*,*j*_ = (*s*(*j*), *s*(*j* + 1), …, *s*(*j* + *m* − 1)). Entropy was calculated as the negative logarithm of the probability that if two template vectors of length *m* have distance less than *r* (i.e., *d*[*s*_*m*,*i*_, *s*_*m*,*j*_] < *r*) then two template vectors of length *m* + 1 also have distance less than *r* (i.e., *d*[*s*_*m*+1,*i*_, *s*_*m*+1,*j*_] < *r*) such as

$$-\ln\frac{F^{m}\left(r\right)}{G^{m}\left(r\right)}$$

where *F*^*m*^(*r*) is the number of template vector pairs having *d*[*s*_*m*+1,*i*_, *s*_*m*+1,*j*_] < *r*, *G*^*m*^(*r*) is the number of template vector pairs having *d*[*s*_*m*,*i*_, *s*_*m*,*j*_] < *r*, and where *d* was the Euclidean distance (Richman & Moorman, 2000) (see Table 1). In our study, *m* = 3, as suggested in two previous studies that computed entropy from resting-state fMRI data in controls (Z. Wang et al., 2014) and in pwMS (Zhou et al., 2016). The parameter *r* was defined as 0.2 *sd*(*s*) where *sd*(*s*) is the standard deviation of the time series (*s*), as recommended by previous work (Tomčala, 2020). An individual’s global entropy was calculated as the average of their regional entropies.

### Mass Univariate Analysis

First, demographics and clinical variables were tested for differences between the three groups (HC, pwMS who had no disability, and pwMS who had disability) using Chi-squared test for qualitative variables and permutation test (David, 2008) for quantitative variables. Second, fractional occupancy of and transition probabilities between brain states and regional entropy were compared between groups via permutation test. *P* values from permutation test were computed as the number of permutations that had a difference in means bigger than the original difference. Group differences were considered significant when *p* < 0.05 after Benjamini–Hochberg (BH) correction (Benjamini & Hochberg, 1995) for multiple comparisons. Finally, the association between average regional (global) entropy and average (global) transition energy was computed using Spearman’s rank correlation, while the association between global entropy and log of lesion volume (transformed to ensure normality) was computed using Pearson’s correlation. All statistical analyses and graphs were performed using R (https://www.r-project.org) version 3.4.4 and Matlab (https://www.mathworks.com/) version R2020a.

### Classification Analysis

Logistic regression with ridge regularization was used to classify (a) HC and pwMS without disability, (b) HC versus pwMS with disability, and (c) pwMS without versus with disability, using demographics/clinical information (age for HC vs. pwMS classification and age and disease duration only for the classification of pwMS subgroups) and vectorized transition energy matrices.

The models were trained with outer and inner loops of k-fold cross-validation (k = 5) to optimize the hyperparameter (*λ*) and test model performance. The folds for both inner and outer loops were stratified to ensure that each fold contained the same proportion of subjects in the two classes as the original dataset. The inner loop (repeated over five different partitions of the training dataset only) optimized the set of hyperparameters that maximized the validation set area under curve (AUC). A model was then fitted using the entire training dataset and the optimal hyperparameters, which was then assessed on the hold-out test set from the outer loop. The outer loop was repeated for 100 different random partitions of the data. The median AUC (over all 5 folds × 100 iterations = 500 test sets) was calculated to assess the performance of the models.

When the data contains class imbalance, models tend to favor the majority class. Due to the class imbalance in our data (66 vs. 33 pwMS with no disability vs. evidence of disability), the oversampling approach synthetic majority oversampling technique (SMOTE) (Chawla et al., 2002) was used to obtain a balanced training dataset during cross-validation. SMOTE compensates for imbalanced classes by creating synthetic examples using nearest neighbor information and has been shown to be among the most robust and accurate methods with which to control for imbalanced data (Santos et al., 2018).

The interpretation of the parameter coefficients from linear models is difficult when there are colinearities in the data (Haufe et al., 2014). To mitigate this problem, we applied the Haufe transform to the model coefficients estimated in each outer loop. Specifically, solving logistic regression with ridge regularization yields model coefficient estimates

$$w_{\text{ridge}}={\left(\Sigma_{x}+\lambda I\right)}^{-1}\;\left(\mu_{x}^{+}-\mu_{x}^{-}\right)$$

where Σ_*x*_ is the covariance matrix of the input variables *x* in the training dataset, *λ* is the hyperparameter of the ridge classifier optimized in the inner loop for the training dataset, *I* is the identity matrix with the same dimension as Σ_*x*_, $\mu_{x}^{+}$, and $\mu_{x}^{-}$ are the average of the input variables for the positive and negative classes in the training dataset. The Haufe transform for these model coefficients is thus

$$\Sigma_{x}{\left(\Sigma_{x}+\lambda I\right)}^{-1}\;\left(\mu_{x}^{+}-\mu_{x}^{-}\right)\text{var}\left(\hat{y}\right)$$

where $\hat{y}$ is the output of the model for the training dataset. The Haufe-transformed model coefficients were averaged over all 5 folds × 100 iterations = 500 training sets, and the result was used to interpret the relationship between TE and the three groups of individuals.

### Data/Code Availability Statement

The de-identified data that support the findings of this study are available upon reasonable request from the corresponding author. The codes that were used to generate the results and figures are publicly available. Please see (a) https://github.com/cerent/MS-NCT/ for the codes that were used for the classification analysis and figures and (b) the paper of Cornblath et al. (2020) for the codes that were used for the clustering and network control theory approach.

## RESULTS

### Patient Characteristics

Table 2 shows the subjects’ demographic and clinical information including sex, age, disease duration, EDSS, MS phenotype, and lesion volume. Unsurprisingly, pwMS with disability were older than both HCs and pwMS without disability (corrected *p* < 0.05) and had a trend toward shorter disease duration compared to pwMS without disability (corrected *p* = 0.161). Sex was not different across any of the groups. Phenotype and disability groups were not independent (corrected *p* < 0.05), where CIS individuals were included in the without disability group and PPMS individuals were included in the with disability group. The MS subgroups did not have a significant difference in lesion volume (corrected *p* value > 0.05).

**Table 2.** Subject demographics and clinical information

Variable	HC (*n* = 19)	pwMS without disability (*n* = 66)	pwMS with disability (*n* = 33)	*P* value (pwMS without disability vs. pwMS with disability)
Age	45 [35.55, 49.50]	40.50 [35, 50] *p* value vs. HC = 0.88	56 [46, 58] *p* value vs. HC = 0.005*	0.0001*
Female (%)	11 (55%)	46 (70%) *p* value vs. HC = 0.41	20 (61%) *p* value vs. HC = 0.96	0.49
Disease duration		10 [7,14.75]	13 [9, 17]	0.161
EDSS		0 [0, 1]	2 [2, 3]	<2.2e−16*
Phenotype		7 CIS, 59 RRMS	28 RRMS, 5 PPMS	<0.001*
Lesion volume (mm^3^)		1,904 [726, 4111]	2,482 [453, 7788]	0.0948

*Note*. Values are presented as median [1st, 3rd quantile] for the continuous variables and as number (percent) for sex. The HC versus pwMS without and with disability as well as the two groups of pwMS were tested for differences; *p* values shown are corrected for multiple comparisons; * indicates significance.

### Brain States and Comparison of Dynamics

**Figure 2.**
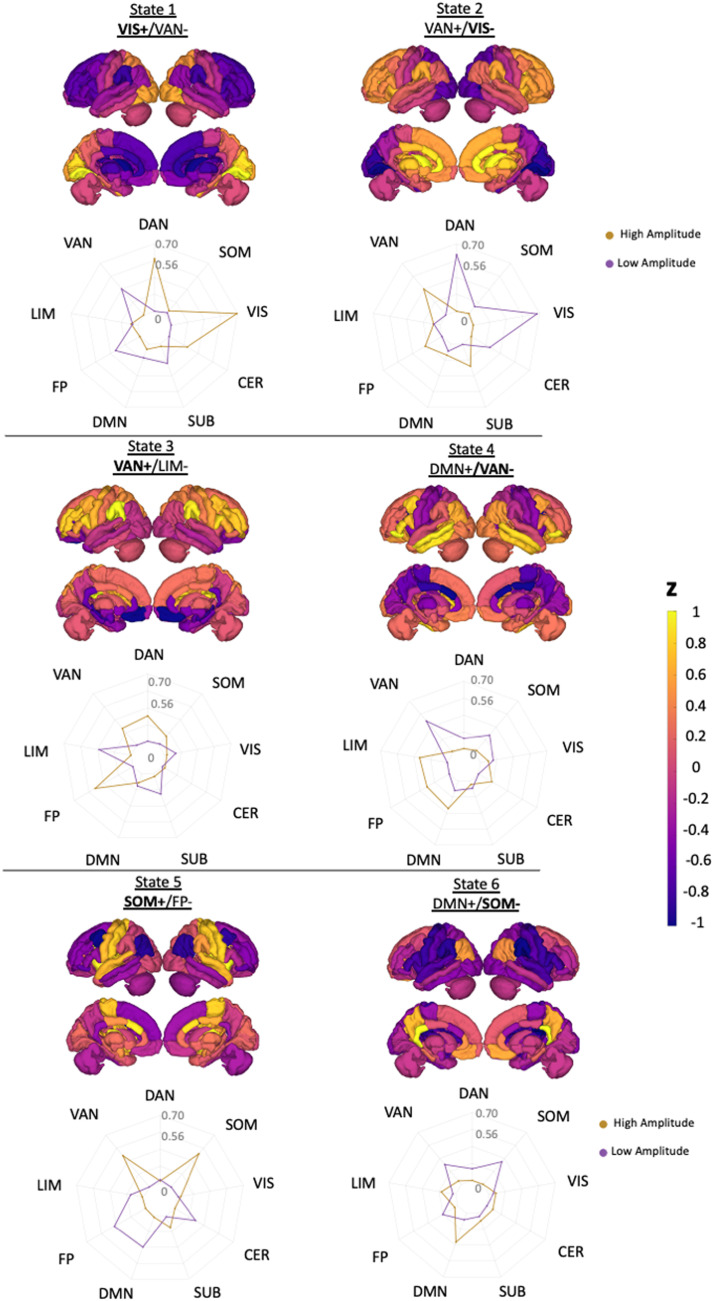
Brain states are visualized using glass brain and radial plots. The radial plots show the mean of the positive and negative brain-state values over the Yeo functional network; DAN = dorsal attention; VAN = ventral attention; LIM = limbic; FP = fronto-parietal; DMN = default-mode network; SUB = subcortex; CER = cerebellum; VIS = visual; SOM = somatomotor. States were named based on the network having the maximum magnitude in the radial plot.

Figure 2 shows the six brain states, consisting of three pairs of anticorrelated states with high/low-amplitude activity in the visual network (VIS+/−), ventral attention network (VAN+/−), and somatomotor network (SOM+/−). The transition probability between dynamic states was not significantly different between HC and any MS subgroup or between MS subgroups (corrected *p* value > 0.05) (see Supporting Information Figure S3). The pwMS subgroups had a trend toward shorter fractional occupancy in the SOM+ and longer fractional occupancy in the VAN+/− and SOM− compared to HC. The pwMS with evidence of disability had a trend toward greater fractional occupancy in the VIS+/−, VAN+, and SOM+ compared to those without disability. However, the differences were not significant for all comparisons (corrected *p* value > 0.05) (see Supporting Information Figure S4).

### Entropy of Brain Activity

**Figure 3.**
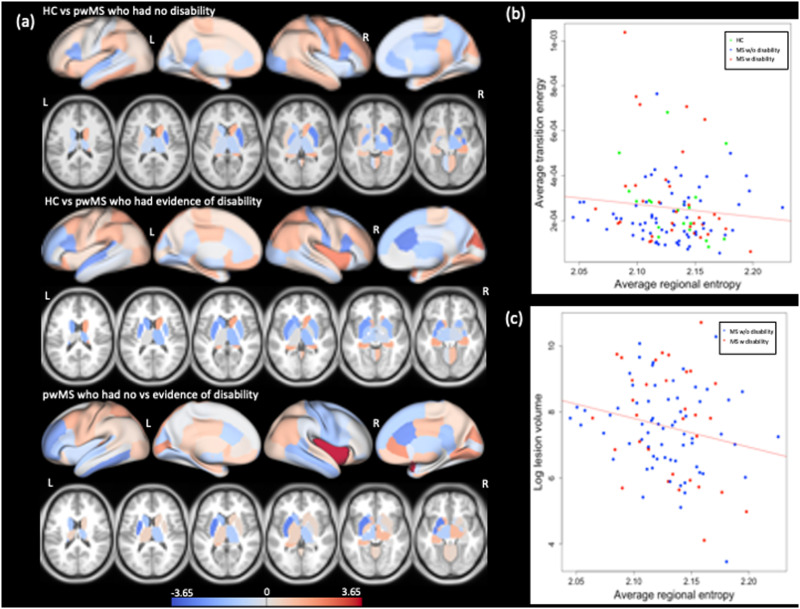
Group difference in entropy and correlation between entropy versus transition energy and lesion load. (A) Group differences in regional entropy between HC versus pwMS without disability (top panel), HC versus pwMS with evidence of disability (middle panel), and between two MS subgroups (bottom panel). The positive *t* statistics presented on the figure indicate greater average entropy in HC compared to MS subgroups as well as greater average entropy in the pwMS with no disability compared to those with evidence of disability. The scatterplot of global entropy versus (A) global transition energy and (B) log of lesion volume (in mm^3^). The entropy and energy were negatively correlated with average transition energy (*r* = −0.17, *p* value = 0.04) and lesion volume (*r* = −0.20, *p* value = 0.03). Green points represent HC (only present in the global energy plot), blue dots represent pwMS without disability, and red dots represent the pwMs with evidence of disability.

Figure 3 shows the *t*-statistics comparing regional entropy between HC and both MS subgroups and between MS subgroups. There was no significant difference in regional entropy between HC and either MS subgroup (corrected *p* value > 0.05). However, entropy in the right insula, which is in the VAN network, was significantly decreased in the pwMS with disability compared to those without disability (corrected *p* value = 0.003). Figure 3 also shows the scatter plot of global entropy and (a) global transition energy (average across all entries in the transition energy matrix) and (b) log of lesion volume. Lower entropy was significantly correlated with greater transition energy (*r* = −0.17, *p* value = 0.04) and greater lesion volume (*r* = −0.20, *p* value = 0.03). The number of state transitions and overall transition energy are inversely related (higher transition energy demand = fewer transitions), so, unsurprisingly, lower entropy was also significantly correlated with fewer overall state transitions (*r* = 0.30, *p* = 0.0003) (see Supporting Information Figure S6).

### Group Classification Using State Transition Energies

**Figure 4.**
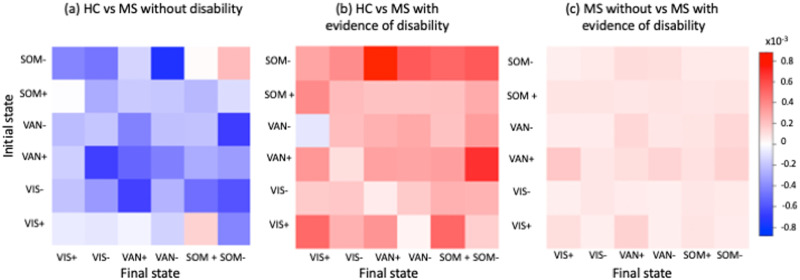
Assessing the relationship between transition energy and disability. Haufe-transformed coefficients for the logistic regression with ridge regularization models classifying HC versus pwMS without disability, HC versus pwMS with disability, and pwMS without disability versus pwMS with disability. VIS = visual; VAN = ventral attention network; SOM = somatomotor.

Figure 4 shows the Haufe-transformed logistic regression model coefficients for classifying HC versus pwMS without disability, HC versus pwMS with disability, and pwMS without versus pwMS with disability; the models had an average AUC of 0.63, 0.62, and 0.62, respectively. Overall, greater transition energy was associated with pwMS with disability compared to both HC and pwMS without disability, while lower transition energy was associated with pwMS without disability compared to HC. Greater transition energies between VAN+ and SOM− were associated with pwMS with disability compared to HC, while smaller transition energies between SOM− and VAN− as well as between VIS− and VAN+ was associated with pwMS without disability compared to HC. Greater transition energy out of VAN+ was most associated with disability in pwMS compared to those without disability.

### Robustness of Results

The analysis was replicated using the cc400 atlas; five optimal states were found: VIS+/−, SOM+/−, and VAN− (see Supporting Information Figure S8), which were overlapping with 4/5 states found with the 86 region atlas. Like the original results, there was no significant difference in fractional occupancy between groups (corrected *p* value > 0.05) (see Supporting Information Figure S9). There was a significant negative correlation between global transition energy and global entropy (*r* = −0.16, *p* value = 0.03), while the correlation between global entropy and lesion load was not significant. Results for the classification model were similar to those obtained with the 86-region atlas include the following: (a) greater overall transition energy was associated with pwMS who had evidence of disability compared to HC and (b) greater transition energies between SOM− and FP+ were associated with pwMS who had disability compared to HC.

## DISCUSSION

In this study, we identified commonly occurring brain states in a group of HC and pwMS individuals, investigated the energy required to transition between them using the NCT approach, and, furthermore, associated these energetic measurements with lesion load and brain activity entropy. Our main findings are (a) there were no group differences (HC vs. pwMS) in the dynamics of the six recurring brain states, made up of high- and low-amplitude activity in visual, ventral attention, and somatomotor networks; (b) lower entropy of global brain activity was associated with greater lesion load and larger global transition energy; and (c) pwMS without disability had decreased transition energies compared to controls, while pwMS with disability had increased transition energy compared to both controls and pwMS without disability. We hypothesized that this latter finding may indicate a shift in the patterns of brain activity in the pwMS without disability that decreases transition energies, but as shift evolves over the disease course, transition energies increase and disability occurs.

A recent study showed HCs had greater brain activity entropy than people with ADHD, and, furthermore, that lower entropy in people with ADHD was related to worse symptom scores (Sokunbi et al., 2013). Additionally, decreased entropy was found in the activity of regions containing a stroke lesion and their contralesional hemisphere homologues (Saenger et al., 2018). Our findings are consistent with these previous findings in that increased lesion volume, and likely a weaker structural backbone that may contribute to increased state transition energy, relates to a decrease in the entropy of brain activity. One study found decreased entropy in the right precentral and left parahippocampus and increased entropy in the left superior temporal, right postcentral, and right transverse temporal in RRMS compared to HC compared to controls, which is largely consistent with our regional results (Zhou et al., 2016). However, the same study showed no significant correlation between overall lesion volume and the averaged entropy computed for each voxel. The difference in our global findings could be due to several factors, including differences in the pwMS cohort characteristics, MRI acquisition and processing differences, and differences in the calculation of entropy (voxel-wise as opposed to region-wise entropy in our study). Finally, greater reductions in global transition energy from the placebo to psychedelic state (in controls) were shown to be associated with larger increases in brain-state entropy (Singleton et al., 2022), which is consistent with our findings of negative correlation between global entropy and transition energy across HC and pwMS.

The brain dynamics have previously been studied by clustering regional fMRI time series directly or sliding-window dynamic functional connectivity in HC (Cohen, 2018; Cornblath et al., 2020; Zalesky et al., 2014) and in patient populations such as stroke (Bonkhoff et al., 2021; L. Wang et al., 2010), schizophrenia (Braun et al., 2021; Damaraju et al., 2014; Rashid et al., 2016), and MS (d’Ambrosio et al., 2019; Rocca et al., 2019; Tozlu et al., 2021a). While many studies investigated the dynamics of sliding-window functional connectivity networks in MS (d’Ambrosio et al., 2019; Rocca et al., 2019; Tozlu et al., 2021a), this approach has some limitations as the window length and shift size need to be selected in a somewhat ad hoc manner. In this study, we use an alternative approach to identify the dynamic brain states in pwMS, for the first time, by clustering the regional time series activity directly. This approach has an advantage compared to other techniques as it is easy to implement, no ad hoc parameter estimation is required, and it uses each of the TRs as an individual data point which is especially helpful with short scans where dFC/FC estimates may be unreliable. NCT and the associated transition energy analysis has been applied previously in HC (Cornblath et al., 2020; Cui et al., 2020; Deng et al., 2022; Gu et al., 2022), in pharmacological states, and in neurological/neuropsychiatric disorders such as schizophrenia and stroke (Braun et al., 2021; Olafson et al., 2022; B. Tang et al., 2022). Using the NCT approach for the pwMS allowed us to jointly capture the effects of MS pathology on SC and co-occurring shifts in brain-state dynamics.

It has been shown in early MS there may be an upregulation of functional activity that initially acts to limit disability, followed by exhaustion of this functional compensation mechanism as disability increases (Tommasin et al., 2018). Previous studies showed these adaptive changes might be most prominent in motor and cognition-related networks, thus limiting clinical measures of disability (Lee et al., 2000) as well as cognitive impairment (Audoin et al., 2005) in MS. Moreover, the extent of the compensatory mechanism was found to be negatively correlated with the degree of tissue damage (Audoin et al., 2005; Rocca et al., 2002), such that more damage was related to decreased compensation. Our study’s finding of overall lower energetic demand in pwMS without disability compared to controls could be reflective of this previously identified compensation mechanism, and our finding of higher energetic demand in pwMS with disability compared to controls could be reflective of the previously identified decrease in compensation mechanism with increased damage (and/or increased damage to SCs).

The SOM and VAN states appeared to be the most important in the three classification models, with largest decreases in transition energy between SOM− and VAN− for pwMS without disability compared to controls and largest decreases in transition energies between SOM− and VAN+ for pwMS with disability compared to controls, and into/out of VAN+ for pwMS without disability versus those with disability. The biggest contributions to the HC versus pwMS with disability classification task were transitions between SOM− and VAN+. Our recent study showed that greater functional connectivity in the SOM and VAN states was associated with pwMS who had no disability compared to those with disability (Tozlu et al., 2021a). In our current study, the SOM− state also had the highest amplitude default mode network (DMN) activity. The VAN and DMN are known to be highly anticorrelated since the DMN is task-negative and the VAN is a task-positive network. Decreased anticorrelation between DMN and attention networks was related to worse behavioral performance in HC (Clare Kelly et al., 2008); moreover, increased functional connectivity between VAN and DMN was observed in pwMS who had cognitive impairment compared to those without cognitive impairment and HC (Huiskamp et al., 2021). Even though we did not have cognitive scores for our cohort, previous studies have identified a strong association between disability and cognitive impairment in MS (Lynch et al., 2005). Our results suggest that dysfunction of the interaction between the VAN and DMN states may result in greater energetic demand between these states in pwMS with evidence of disability compared to the other groups. Finally, our results also showed that greater transition energy between SOM+ and VIS− was associated with pwMS who had disability. The most frequently observed disturbances are the motor and visual impairments in pwMS, which are reflected in worse (higher) EDSS (Fuhr et al., 2001; Jasse et al., 2013). Our study shows, for the first time, greater energetic demand to transition between the motor and visual states compared to HC is associated with disability in MS.

### Limitations

One of the limitations of the study was in the quality of the MRI collected, as the fMRI acquisition time was relatively short and had a longer TR (6 min, TR = 2.3 s) and the dMRI acquisition had only a single b-value of 800 and only 55 directions. A longer fMRI acquisition may result in finding other, less prevalent, dynamic states. However, Cornblath et al. (2020) applied the same clustering method to 15-min fMRI scans and found five optimal states that all had an appearance rate of approximately 2 per minute; thus, we believe our relatively shorter scan still represents the underlying state space well. Another limitation was the relatively low number of HCs compared to pwMS, which could result in an imbalance of the dynamic states. We used a permutation test when comparing HC and pwMS to minimize the effect of this group size imbalance. Larger lesion volume in some subjects may negatively impact coregistration to MNI space, which is where the FCs were calculated. In our cohort, lesions were generally on the small side, but we did visually inspect the MNI coregistration for quality and adjust if needed. Another limitation of the study was that most of the MS patients used in our study were of the RRMS phenotype, therefore the results may mostly represent disease processes within this subtype. A future study that includes a similar number of all MS phenotypes such as CIS, RRMS, PPMS, and SPMS is needed to capture the variability due to different level of disability in MS. Lastly, this study was cross-sectional; future studies will investigate how brain dynamics change over time and relate to physical and cognitive decline.

### Conclusion

This is the first study using NCT to investigate how dynamic brain states and their associated transition energies change in MS and, furthermore, to associate brain activity entropy with lesion load and NCT-derived brain-state transition energies. As hypothesized, we found that global brain activity entropy decreased with increasing lesion load and increasing transition energies. Greater overall transition energies were associated with pwMS who had evidence of disability compared to both HC and pwMS without disability, while decreased overall transition energies were associated with pwMS without disability compared to the other two groups. Transition energies in ventral attention and somatomotor networks largely drove the group classifications. This study, through NCT, sheds light on a possible mechanism of how MS-related damage to the brain’s structural backbone can impact brain activity dynamics, entropy and energetics.

## SUPPORTING INFORMATION

Supporting information for this article is available at https://doi.org/10.1162/netn_a_00292.

## AUTHOR CONTRIBUTIONS

Ceren Tozlu: Data curation; Investigation; Visualization; Writing – original draft. Sophie Card: Validation. Keith Jamison: Data curation; Writing – review & editing. Susan Gauthier: Conceptualization; Data curation; Supervision; Writing – review & editing. Amy Kuceyeski: Conceptualization; Supervision; Writing – review & editing.

## FUNDING INFORMATION

Amy Kuceyeski, NIH, Award ID: NS104634-01. Amy Kuceyeski, NIH, Award ID: NS102646-01A1. Susan Gauthier, Weill Cornell Medicine, Award ID: TR000456-06. Ceren Tozlu, National Multiple Sclerosis Society (https://dx.doi.org/10.13039/100000890), Award ID: FG-2008-36976.

## TECHNICAL TERMS

Functional connectivity: Temporal coactivation of the brain’s neural activity between different brain regions of interest.
Network control theory: An approach to identify the brain’s energetic landscape using the structural connectome and patterns of brain activity over time.
Brain state: The recurring patterns of the brain’s dynamic neural activity.
Entropy: A measure indicating the complexity and predictability of a dynamic system.
Transition energy: The minimum energy required to transition between pairs of brain states or to remain in the same state over some time.
Transition probability: The probability that a brain switches to a different state or remains in the same state (i.e., persistence probability) at the next time point.
Fractional occupancy: The number of TRs (repetition time) assigned to each cluster out of the total number of TRs.
EDSS: Expanded Disability Status Scale that measures the disability level in people with multiple sclerosis. EDSS ranges between 0 and 10, where 0 means that the subject does not have a disability and 10 means death due to multiple sclerosis.
Structural connectivity: Strength of the anatomical connections between different brain regions of interest.
